# Supplementary material for: Predicting visual function by interpreting a neuronal wiring diagram
Source: Nature. 2024 Oct 2;634(8032):113–23. doi: 10.1038/s41586-024-07953-5 (PMC11446822; doi:10.1038/s41586-024-07953-5)
Supplement: Supplementary file 6 — CRF and ERF predictions for individual TmY4 and TmY9 cells. Analogous to Supplementary Data 3, but for TmY target types. Shown are the top four monosynaptic pathways, the strongest pathway passing through each of the top ten intermediary types (ranking from Extended Data Fig. 7), and the trisynaptic pathway Tm1–TmY–Dm3–TmY (see the section entitled Prediction of spatial normalization). [file 41586_2024_7953_MOESM6_ESM.zip › DataS4/TmY9qΓèÑ/720575940605710112.pdf]

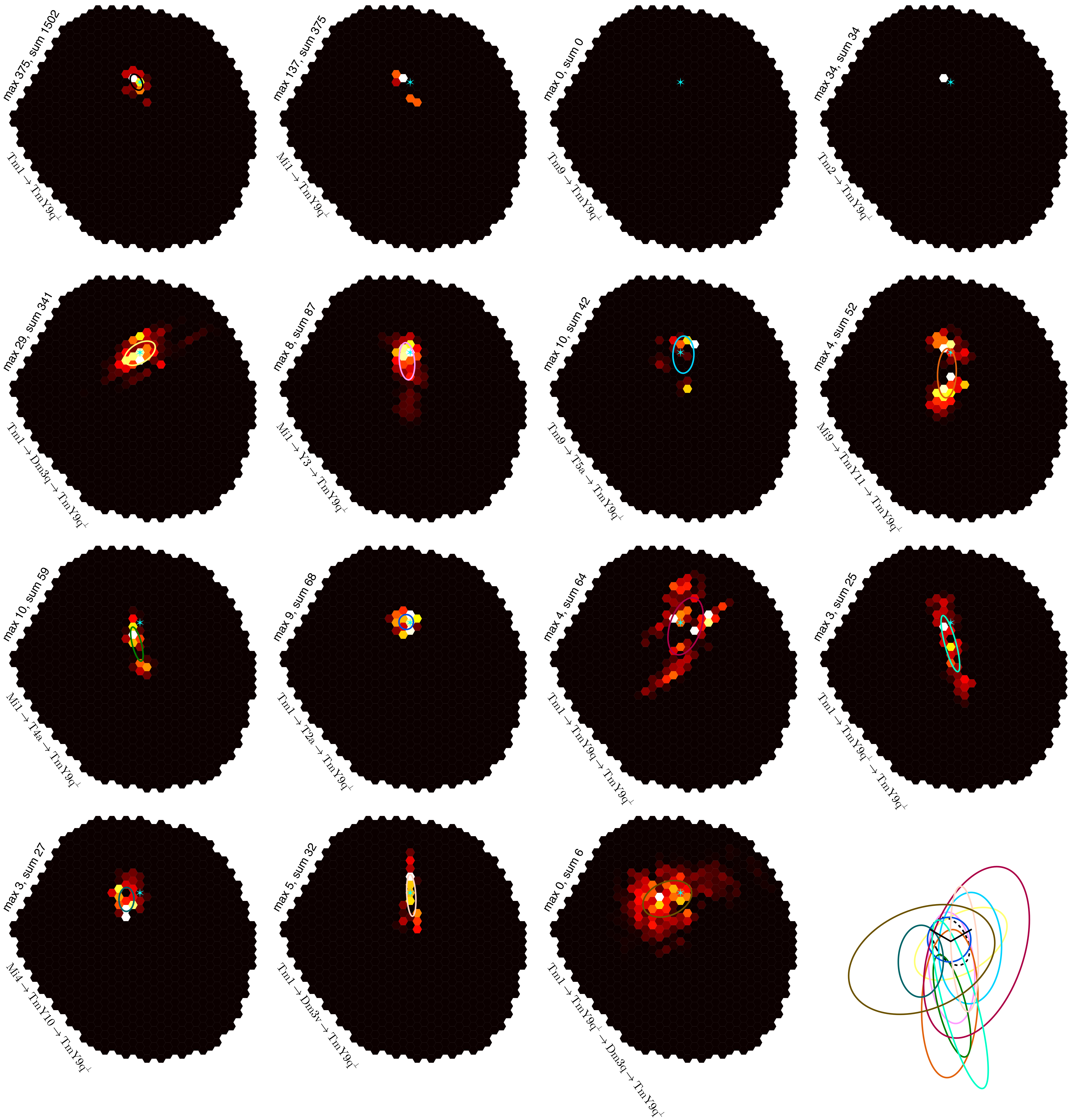
max 375, sum 1502
Tm1 → TmY9q⊥
max 137, sum 375
Mi1 → TmY9q⊥
max 0, sum 0
Tm9 → TmY9q⊥
max 34, sum 34
Tm2 → TmY9q⊥
max 29, sum 341
Tm1 → Dm3q → TmY9q⊥
max 8, sum 87
Mi1 → Y3 → TmY9q⊥
max 10, sum 42
Tm9 → T5a → TmY9q⊥
max 4, sum 52
Mi9 → TmY11 → TmY9q⊥
max 10, sum 59
Mi1 → T4a → TmY9q⊥
max 9, sum 68
Tm1 → T2a → TmY9q⊥
max 4, sum 64
Tm1 → TmY9q → TmY9q⊥
max 3, sum 25
Tm1 → TmY9q⊥ → TmY9q⊥
max 3, sum 27
Mi4 → TmY10 → TmY9q⊥
max 5, sum 32
Tm1 → Dm3v → TmY9q⊥
max 0, sum 6
Tm1 → TmY9q⊥ → Dm3q → TmY9q⊥
